# The impact of perivesical lymph node metastasis on clinical outcomes of bladder cancer patients undergoing radical cystectomy

**DOI:** 10.1186/s12894-019-0507-z

**Published:** 2019-08-16

**Authors:** Meenal Sharma, Takuro Goto, Zhiming Yang, Hiroshi Miyamoto

**Affiliations:** 1 Department of Pathology and Laboratory Medicine, University of Rochester Medical Center, Rochester, NY USA; 2 James P. Wilmot Cancer Institute, University of Rochester Medical Center, Rochester, NY USA; 3 Department of Urology, University of Rochester Medical Center, Rochester, NY USA

**Keywords:** Cancer-specific survival, Mortality, Pelvic lymph node metastasis, Progression-free survival, Staging, Urothelial carcinoma

## Abstract

**Background:**

Perivesical lymph nodes (PVLNs) are occasionally isolated during grossing of cystectomy specimens. However, the prognostic implications of the involvement of PVLNs in bladder cancer patients, especially those with comparisons to pN0 disease, remain poorly understood.

**Methods:**

A retrospective review identified 115 radical cystectomy cases where PVLNs had been histologically assessed. These cases were then divided into 4 groups – Group 1 (*n* = 76): PVLN-negative/other pelvic lymph node (non-PVLN)-negative; Group 2 (*n* = 5): PVLN-positive/non-PVLN-negative; Group 3 (*n* = 17): PVLN-negative/non-PVLN-positive; and Group 4 (*n* = 17): PVLN-positive/non-PVLN-positive.

**Results:**

pT stage at cystectomy was significantly higher in Group 3 (*P* = 0.013), Group 4 (*P* < 0.001), Groups 2 and 4 (*P* < 0.001), or Groups 2–4 (*P* < 0.001) than in Group 1. However, the number of positive PVLNs (mean: 1.8 vs. 2.1; *P* = 0.718) or the rate of extracapsular extension in the PVLNs (40% vs. 65%, *P* = 0.609) was not significantly different between Group 2 and Group 4. Kaplan-Meier analysis and log-rank test revealed significantly (*P* < 0.05) higher risks of disease progression (Group 3/Group 4), cancer-specific mortality (Group 2/Group 3/Group 4), and overall mortality (Group 4), compared with Group 1. Multivariate analysis further showed metastasis to both PVLN and non-PVLN (Group 4), PVLN (Groups 2 and 4), or PVLN and/or non-PVLN (Groups 2–4) as an independent prognosticator for cancer-specific mortality and overall survival. There were also insignificant (*P* = 0.096) and significant (*P* = 0.036) differences in cancer-specific survival and overall survival, respectively, between Group 3 versus Group 4, and the trend of the latter was confirmed by subset multivariate analysis (hazard ratio = 3.769; *P* = 0.099).

**Conclusions:**

Worse prognosis was observed in bladder cancer patients with isolated PVLN metastasis (vs. pN0 disease especially for cancer-specific survival), PVLN metastasis with or without non-PVLN metastasis (vs. pN0 disease), and concurrent PVLN and non-PVLN metastases (vs. PVLN-negative/non-PVLN-positive disease especially for overall survival). These findings indicate the importance of thorough histopathological assessment of PVLNs in radical cystectomy specimens.

## Background

Urinary bladder cancer, which is mostly a urothelial carcinoma, is one of the most frequently diagnosed neoplasms worldwide [1]. Muscle-invasive disease with which approximately 30% of patients initially present accounts for the majority of mortalities associated with bladder cancer [2, 3]. Additionally, it has been well documented that metastasis to the regional lymph node(s) represents a critical prognostic factor in patients with bladder cancer. Meanwhile, radical cystectomy usually with pelvic lymph node dissection remains the mainstay of treatment for locally advanced bladder cancer.

Previous studies have addressed the clinical impact of the extent and boundaries of lymph node dissection during radical cystectomy on patient outcomes [4–7]. By contrast, the prognostic implications of perivesical lymph node (PVLN) involvement by bladder cancer metastasis remain far from being fully understood. It has been variably reported that PVLNs are isolated in 0–46% of patients via grossing and histological assessment of cystectomy specimens, presumably dependent on surgical technique and tissue processing [8–13]. Metastasis to the PVLNs has also been found in 3–21% of the cystectomy cases for bladder cancer in which PVLNs are identified [8, 11–13]. Importantly, in the latest edition of the American Joint Committee on Cancer (AJCC) TNM staging for bladder cancer (8th Edition implemented on January 1, 2018) [14], PVLN metastasis was definitively classified as N1 (single node involvement) or N2 (multiple node involvement). However, no recent studies have assessed the clinical significance of PVLN involvement in patients with bladder cancer, especially with comparisons to N0 disease. We here comparatively studied the outcome of groups of patients with or without positive PVLN and/or non-PVLN who underwent radical cystectomy and pelvic lymphadenectomy for bladder cancer.

## Methods

We searched our Surgical Pathology database for radical cystectomy/cystoprostatectomy cases performed between July 2004 and March 2019. Of these, 115 primary bladder cancer cases (93 males and 22 females; age range: 25–88 years; mean age: 68.5 years; median age: 69 years) where PVLNs had been histologically assessed were identified. All these 115 patients underwent pelvic lymph node dissection. The cystectomy specimens were grossly reviewed for the presence of PVLNs and processed primarily by pathology assistants and/or pathology residents who were supervised by attending pathologists. Histologically, an aggregate of lymphoid tissue at least partially encapsulated was defined as a lymph node. Clearing techniques or solvents, as well as special stains, for isolating PVLNs were not used. We also retrieved clinical and histopathological findings as well as follow-up data (median: 24 months; 5 cases lost to follow-up and 4 recent cases) from all 115 patients. Neoadjuvant systemic chemotherapy prior to cystectomy or adjuvant systemic chemotherapy/immunotherapy following cystectomy was performed in 21 or 25 patients, respectively.

Data were analyzed, using the Student’s *t*-test for continuous variables and the Fisher’s exact test for non-continuous variables. The rates of progression-free survival, cancer-specific survival, and overall survival were calculated by the Kaplan-Meier method, and comparison was made by log-rank test. Tumor progression was defined as the development of recurrent or metastatic tumors after cystectomy. In addition, the Cox proportional hazards model was used to determine statistical significance of prognostic factors in a multivariate setting. *P* values less than 0.05 were considered to be statistically significant.

## Results

We analyzed 115 radical cystectomy cases where PVLNs (range: 1–14; mean: 2.3; median: 2) were histologically assessed. Twenty-two (19%) and 34 (30%) patients had metastases to the PVLNs and other pelvic lymph nodes (non-PVLNs), respectively. Of the latter cases, 3 had pN3 disease, but none had isolated metastasis to the common iliac lymph nodes. Seventeen (15%) patients showed concurrent metastases to both PNLN and non-PVLN. For further analyses, these cases were divided into 4 groups based on the status of lymph node metastasis – Group 1 (*n* = 76): PVLN-negative/non-PVLN-negative; Group 2 (*n* = 5): PVLN-positive/non-PVLN-negative; Group 3 (*n* = 17): PVLN-negative/non-PVLN-positive; and Group 4 (*n* = 17): PVLN-positive/non-PVLN-positive.

Table 1 summarizes the clinicopathological features of the 4 cohorts of patients. Clinical stage prior to neoadjuvant chemotherapy and subsequent cystectomy was significantly higher (≥III) in Group 4 (35%, *P* = 0.021), Groups 2 and 4 (29%, *P* = 0.039), or Groups 2–4 (28%, *P* = 0.034) than in Group 1 (11%). Similarly, pT stage at cystectomy was significantly higher (≥pT3) in Group 3 (71%, *P* = 0.013), Group 4 (94%, *P* < 0.001), Groups 2 and 4 (86%, *P* < 0.001), or Groups 2–4 (79%, *P* < 0.001) than in Group 1 (36%). In addition, adjuvant therapy was more often administered in Group 4 (47%, *P* = 0.006), Groups 2 and 4 (41%, *P* = 0.010), or Groups 2–4 (36%, *P* = 0.010) than in Group 1 (14%). However, there were no significant differences in age, sex, neoadjuvant chemotherapy, histology [conventional urothelial carcinoma vs. variants including squamous differentiation (*n* = 14), glandular differentiation or adenocarcinoma (*n* = 3), small cell carcinoma (*n* = 3), and micropapillary (*n* = 5), nested (*n* = 2), or sarcomatoid (*n* = 3) feature], and surgical margin status between groups. Meanwhile, the number of positive PVLNs and the size of largest tumor focus in the PVLN were not significantly different between Groups 2 and 4 (Table 2). The rate of extracapsular extension in positive PVLNs in Group 4 (65%) was higher than that in Group 2 (40%), although the difference was not statistically significant (*P* = 0.609).

**Table 1:** Clinicopathological features of patients undergoing radical cystectomy and pelvic lymphadenectomy

	Group 1	Group 2	Group 3	Group 4	*P* value
	PVLN(−)/non-PVLN(−)	PVLN(+)/non-PVLN(−)	PVLN(−)/non-PVLN(+)	PVLN(+)/non-PVLN(+)	
No. of patients	76	5	17	17	
Age (mean ± SD, years)	67.6 ± 12.5	73.8 ± 11.0	70.8 ± 8.9	68.9 ± 9.7	> 0.1^a^
Sex					> 0.1^a^
Male	60 (79%)	4 (80%)	13 (76%)	16 (94%)	
Female	16 (21%)	1 (20%)	4 (24%)	1 (6%)	
Clinical stage (prior to cystectomy)					0.021^b^ (G1 vs G4); 0.039^b^ (G1 vs G2&G4); 0.034^b^ (G1 vs G2-G4)
≤ II	61 (80%)	4 (80%)	13 (76%)	10 (59%)	
≥ III	8 (11%)	1 (20%)	4 (24%)	6 (35%)	
Unknown	7 (9%)	0 (0%)	0 (0%)	1 (6%)	
Neoadjuvant chemotherapy					> 0.1^a^
No	65 (86%)	4 (80%)	12 (71%)	13 (76%)	
Yes	11 (14%)	1 (20%)	5 (29%)	4 (24%)	
Histology					> 0.1^a^
Conventional	56 (74%)	5 (100%)	14 (82%)	10 (59%)	
Variants	20 (26%)	0 (0%)	3 (18%)	7 (41%)	
pT stage					0.013 (G1 vs G3); < 0.001 (G1 vs G4); < 0.001 (G1 vs G2&G4); < 0.001 (G1 vs G2-G4)
≤ 2	49 (64%)	2 (40%)	5 (29%)	1 (6%)	
≥ 3	27 (36%)	3 (60%)	12 (71%)	16 (94%)	
Surgical margin					> 0.1^a^
No	71 (93%)	4 (80%)	17 (100%)	14 (82%)	
Yes	5 (7%)	1 (20%)	0 (0%)	3 (18%)	
Adjuvant therapy					0.006 (G1 vs G4); 0.010 (G1 vs G2&G4); 0.010 (G1 vs G2-G4)
No	65 (86%)	4 (80%)	12 (71%)	9 (53%)	
Yes	11 (14%)	1 (20%)	5 (29%)	8 (47%)	

*Abbreviation*: *PVLN* perivesical lymph node

^a^all comparisons performed between two groups

^b^≤ II vs ≥ III

**Table 2:** Characteristics of PVLNs

	Group 2	Group 4	*P* value
	PVLN(+)/non-PVLN(−)	PVLN(+)/non-PVLN(+)	
No. of patients	5	17	
No. of PVLN [mean / median (range)]	5.0 / 4 (1–14)	3.1 / 2 (1–12)	0.472
No. of positive PVLN [mean / median (range)]	1.8 / 1 (1–4)	2.1 / 1 (1–12)	0.718
Largest tumor focus [mean / median (range), cm]	0.8 / 0.8 (0.2–1.1)	0.7 / 0.8 (0.1–2.4)	0.989
Extracapsular extension	2 (40%)	11 (65%)	0.609

*Abbreviation*: *PVLN* perivesical lymph node

**Fig. 1:**
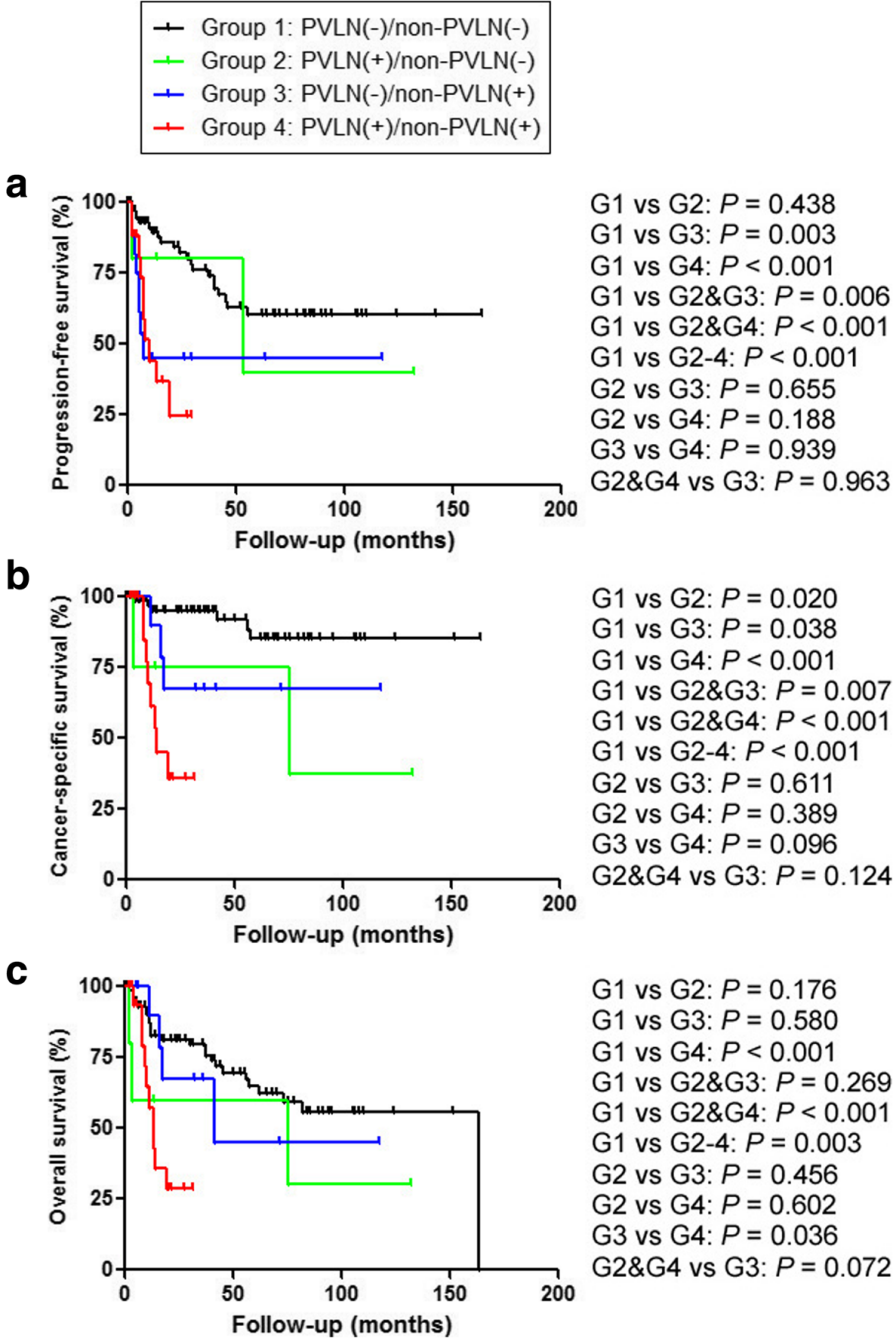
Progression-free survival (**a**), cancer-specific survival (**b**), or overall survival (**c**) in bladder cancer patients undergoing radical cystectomy and pelvic lymphadenectomy according to the status of PVLN and non-PVLN metastases (i.e. Groups 1–4). Comparisons between two groups were made by log-rank test

We further investigated possible associations between the status of PVLN/non-PVLN metastasis and patient outcomes after radical surgery. The median follow-up periods for overall survival in alive patients were: 50 (Group 1); 72.5 (Group 2); 6 (Group 3); and 20 (Group 4) months. Kaplan-Meier analysis coupled with log-rank test revealed significantly worse prognosis in Group 2 (cancer-specific survival), Group 3 (disease-free/cancer-specific survival), and Group 4 (progression-free/cancer-specific/overall survival), compared with Group 1 (Fig. 1). Significantly higher risks of disease progression/mortality were also seen in patients with lymph node metastasis (Groups 2, 3, and 4), either PVLN or non-PLVN metastasis (Groups 2 and 3; except overall survival), or PVLN metastasis (Groups 2 and 4), compared to those with pN0 disease (Group 1). Interestingly, there were insignificant (*P* = 0.096) and significant (*P* = 0.036) differences in cancer-specific survival and overall survival, respectively, between Groups 3 versus 4. Similarly, insignificantly worse overall survival (*P* = 0.072) was seen in PVLN-positive cases (Groups 2 and 4), compared with Group 3. No statistically significant differences in patient outcomes between Groups 2 versus 3 and Groups 2 versus 4 were observed. Meanwhile, neoadjuvant and adjuvant therapies were associated with significantly lower rates of progression-free survival and progression-free/cancer-specific survival, respectively (see Table 3).

**Table 3:** Univariate and multivariate analyses of survival in patients undergoing radical cystectomy and pelvic lymphadenopathy

	Progression-free survival				Cancer-specific survival				Overall survival			
	Univariate		Multivariate		Univariate		Multivariate		Univariate		Multivariate	
	HR (95% CI)	*P* value	HR (95% CI)	*P* value	HR (95% CI)	*P* value	HR (95% CI)	*P* value	HR (95% CI)	*P* value	HR (95% CI)	*P* value
Lymph node status												
Group 1; PVLN(−)/non-PVLN(−)	1 (reference)		1 (reference)		1 (reference)		1 (reference)		1 (reference)		1 (reference)	
Group 2; PVLN(+)/non-PVLN(−)	2.086 (0.325–13.38)	0.438	0.559 (0.117–2.665)	0.465	33.28 (1.719–644.3)	0.020	3.570 (0.525–24.27)	0.193	3.224 (0.593–17.54)	0.176	1.977 (0.496–7.874)	0.334
Group 3; PVLN(−)/non-PVLN(+)	6.191 (1.869–20.51)	0.003	1.523 (0.570–4.068)	0.401	8.894 (1.128–70.34)	0.038	3.166 (0.630–15.93)	0.162	1.403 (0.423–4.657)	0.580	1.657 (0.509–5.398)	0.402
Group 4; PVLN(+)/non-PVLN(+)	22.62 (6.259–81.76)	< 0.001	1.448 (0.571–3.671)	0.435	142.6 (25.61–794.0)	< 0.001	7.667 (1.894–31.05)	0.004	12.36 (3.751–40.72)	< 0.001	3.474 (1.376–8.771)	0.008
Age (years)												
≤ 69	1 (reference)		NA		1 (reference)		NA		1 (reference)		NA	
≥ 70	1.237 (0.662–2.313)	0.505			1.074 (0.431–2.675)	0.878			1.581 (0.847–2.951)	0.150		
Sex												
Male	1 (reference)		1 (reference)		1 (reference)		1 (reference)		1 (reference)		1 (reference)	
Female	0.601 (0.279–1.297)	0.195	1.066 (0.379–2.996)	0.904	0.289 (0.091–0.917)	0.035	< 0.001 (0-Infinity)	0.997	0.543 (0.248–1.191)	0.128	0.503 (0.170–1.486)	0.214
Neoadjuvant chemotherapy												
No	1 (reference)		1 (reference)		1 (reference)		1 (reference)		1 (reference)		1 (reference)	
Yes	2.994 (1.117–8.028)	0.029	2.484 (1.036–5.952)	0.041	0.865 (0.214–3.504)	0.840	0.615 (0.121–3.134)	0.559	0.636 (0.243–1.664)	0.356	0.507 (0.150–1.710)	0.274
Histology												
Conventional	1 (reference)		1 (reference)		1 (reference)		1 (reference)		1 (reference)		1 (reference)	
Variants	0.951 (0.441–2.050)	0.898	0.475 (0.183–1.234)	0.126	1.525 (0.490–4.746)	0.466	1.723 (0.488–6.083)	0.398	2.039 (0.947–4.386)	0.069	1.442 (0.614–3.391)	0.401
pT stage												
≤ 2	1 (reference)		1 (reference)		1 (reference)		1 (reference)		1 (reference)		1 (reference)	
≥ 3	5.547 (2.917–10.55)	< 0.001	5.829 (2.238–15.18)	< 0.001	6.293 (2.494–15.88)	< 0.001	2.716 (0.623–11.87)	0.184	3.392 (1.796–6.406)	< 0.001	2.057 (0.842–5.024)	0.113
Surgical margin												
Negative	1 (reference)		1 (reference)		1 (reference)		1 (reference)		1 (reference)		1 (reference)	
Positive	7.764 (2.086–28.89)	0.002	1.766 (0.681–4.578)	0.242	4.713 (0.752–29.53)	0.098	2.640 (0.588–11.86)	0.205	4.051 (1.148–14.30)	0.030	2.472 (0.949–6.443)	0.064
Adjuvant therapy												
No	1 (reference)		1 (reference)		1 (reference)		1 (reference)		1 (reference)		1 (reference)	
Yes	5.929 (2.700–13.02)	< 0.001	1.867 (0.891–3.914)	0.098	5.372 (1.835–15.73)	0.002	1.205 (0.429–3.387)	0.724	1.365 (0.644–2.896)	0.417	0.619 (0.289–1.324)	0.216

*Abbreviations*: *HR* hazard ratio, *CI* confidence interval, *PVLN* perivesical lymph node, *NA* not assessed

To determine whether the involvement of PVLNs and/or non-PVLNs was an independent prognosticator in bladder cancer patients undergoing radical cystectomy, multivariate analysis was performed with the Cox model for all the variables assessed for univariate analysis except age showing no prognostic significance in any comparisons. Metastasis to both PVLN and non-PVLN (Group 4) was independently associated with poor cancer-specific survival and overall survival (Table 3). Patients undergoing neoadjuvant and adjuvant therapies were also significantly (*P* = 0.041) and insignificantly (*P* = 0.098), respectively, associated with disease progression. Multivariate analysis in subgroups of patients further showed that metastasis to PVLN [Groups 2 and 4; hazard ratio (HR) = 5.898, 95% confidence interval (CI) = 1.424–24.42; *P* = 0.014 for cancer-specific survival; HR = 2.690, 95% CI = 1.109–6.525, *P* = 0.029 for overall survival] or either PVLN or non-PVLN or both (Groups 2–4; HR = 5.053, 95% CI = 1.474–17.32, *P* = 0.010 for cancer-specific survival; HR = 2.493, 95% CI = 1.130–5.501, *P* = 0.024 for overall survival), compared with no metastatic disease (Group 1), was an independent predictor. In addition, those with PVLN-positive/non-PVLN-positive disease (Group 4) tended to have worse overall survival (HR = 3.769; 95% CI = 0.777–18.28; *P* = 0.099), but not cancer-specific survival (HR = 3.195; 95% CI = 0.637–16.15; *P* = 0.160), compared to those with PVLN-negative/non-PVLN-positive disease (Group 3).

## Discussion

Several studies have addressed the role of assessing the PVLNs histopathologically in radical cystectomy specimens [8–13]. In only two of the studies [8, 13], the prognostic significance of PVLN metastasis from bladder cancer has been simultaneously investigated. To the best of our knowledge, however, no studies have definitively compared the outcomes of bladder cancer patients with N0 disease versus isolated PVLN metastasis. Thus, the primary aim of the present study was to determine the impact of PVLN involvement on oncologic outcomes of patients who underwent radical cystectomy for bladder cancer.

In a study by Bella et al. [8], PVLN was isolated in 32 (16%) of 198 cases undergoing radical cystectomy and pelvic lymphadenectomy for clinically organ-confined bladder urothelial carcinoma, and metastasis to the PVLN was seen in 14 of the patients. Outcome analysis further showed that overall survival (*P* = 0.002), disease-specific survival (*P* = 0.013), and disease-free survival (*P* < 0.001) were significantly worse in patients with PVLN-positive disease than in those with PVLN-negative disease and that PVLN metastasis was an independent predictor of overall mortality (*P* = 0.016) or disease-specific mortality (*P* = 0.025). However, each of the PVLN-positive or PVLN-negative cohort included both other pelvic lymph node positive and negative cases. Meanwhile, significant differences in overall survival (*P* = 0.001), disease-specific survival (*P* = 0.010), and disease-free survival (*P* = 0.023) between metastatic cases to other pelvic lymph node(s) with versus without PVLN involvement were observed. In a more recent study [13], Hu et al. identified the PVLN in 936 (46%) of 2017 tissue specimens from radical cystectomy/pelvic lymphadenectomy (performed in 1971–2009) in 197 (10%) of which metastatic carcinoma was present. On univariate analysis, concurrent metastases to the PVLN and other pelvic lymph node (*n* = 96) were associated with significantly worse recurrence-free survival or overall survival, compared with metastasis only to the PVLN (*n* = 101) or other pelvic lymph node (*n* = 268) in the entire cohort of patients, whereas the associations of survival of the concurrent metastases group (*n* = 43) with that of the other lymph node metastasis only group (*n* = 110), but not with that of the isolated PVLN metastasis group (*n* = 12), were statistically significant in the contemporary subset (2002–2009 cases). Similarly, multivariate analysis in the entire patients (*n* = 465) showed significantly worse prognosis of concurrent PVLN/other lymph node metastases, but not isolated PVLN metastasis, compared with other lymph node metastasis only/PVLN-negative cases. These two studies might have contributed to the definitive classification of PVLN involvement as N1/N2 in the current AJCC staging for bladder cancer [14]. In accordance with these findings [8, 13], we found significantly or marginally worse prognosis in bladder cancer patients with PVLN metastasis, compared to those with PVLN-negative/non-PVLN-positive disease (i.e. Groups 4 vs. 3, Groups 2 & 4 vs. 3), although a slightly higher proportion of patients in Group 4 showed unfavorable histopathological findings, including variant histology, locally advanced stage (≥pT3), and positive surgical margin, compared with those in Group 3. Of note, isolated PVLN metastasis (Group 2) was associated with a significantly higher risk of cancer-specific mortality, compared with pN0 disease (Group 1), although it was found not to be an independent factor when analyzed in a multivariate setting. Additionally, as expected, PVLN metastasis (Groups 2 and 4), as well as PVLN and/or non-PVLN metastasis (Groups 2–4), was found to be an independent predictor of cancer-specific mortality or overall survival. Further validation studies with larger cohorts, including patients with isolated PVLN metastasis as well as no PVLN/non-PVLN metastasis, are thus warranted. It may also be interesting to assess the relationship between the location of positive PVLNs and patient outcomes.

The numbers of lymph nodes histopathologically assessed have been shown to have prognostic implications in bladder cancer patients undergoing radical cystectomy and pelvic lymphadenectomy and even in those with distant metastasis [15, 16]. Nonetheless, the count of pelvic lymph nodes is critically dependent on not only the extent of their dissection or surgical technique but also tissue processing at surgical pathology. Similarly, varied numbers of the PVLNs isolated from cystectomy specimens have been reported in different studies [8–13]. Then, maximum effort to isolate the PVLNs is particularly important when grossing radical cystectomy specimens. Meanwhile, in a study using tissues from lymphadenectomy during radical cystectomy [17], no metastasis was found in considerable numbers of additional lymph nodes that were not grossly identified but were isolated via submission of the entire fatty specimens for histological examination.

Classical understanding of lymphatic drainage of bladder cancer is that positive primary lymph nodes, including obturator, internal and external iliac, and sacral nodes, as well as PVLN in a subset of patients [18], drain into the common iliac region and subsequently lead to distant metastasis, as skip metastases are extremely rare [9, 19]. It has been further hypothesized that bladder cancer drainage within the regional lymph nodes is bidirectional and that some patients have lymphatic drainage from the PVLN to lymphatics other than the primary regional nodes described above [13]. These may explain why bladder cancer patients with PVLN-positive and other pelvic lymph node-positive disease show poorer outcomes than those with PVLN-negative and other pelvic lymph node-positive disease.

There are several limitations in our investigation. First, due to its retrospective design, the present study is subject to potential selection bias. In particular, our database search was solely dependent on the diagnosis of “PVLN” included in the pathology reports of radical cystectomy cases. Pathologists at our institution might not have always reported the presence of PVLNs even if they were found. Moreover, there was no standard protocol for identifying the PVLNs in radical cystectomy specimens. Therefore, we might have missed a considerable number of cases with PVLN. Second, the number of the isolated PVLN metastasis group (Group 2) is relatively small, which may especially affect statistical analyses. Significantly worse prognosis in patients with PVLN metastasis (Groups 2 and 4; *n* = 22) might also have been principally due to that in patients with concurrent PVLN and non-PVLN metastasis (Group 4; *n* = 17). Third, at least 4 surgeons performed radical cystectomy and pelvic lymphadenectomy for our patient cohort, and their surgical techniques, including the extent of node dissection, might thus have varied. Despite the limitations of this study, however, our data showing considerable or no significant differences in patient outcomes between the PVLN-negative/other node-positive and PVLN-positive/other node-positive groups (i.e. Groups 3 vs. 4) or between the PVLN-positive/other node-negative and PVLN-negative/other node-positive groups (i.e. Groups 2 vs. 3), respectively, are consistent with previous observations in a larger-scale study [13]. More strikingly, we are likely the first to demonstrate that patients with isolated PVLN metastasis (Group 2) had a significantly higher risk of cancer-specific mortality, compared to those with pN0 disease (Group 1), at least after definitive classification of PVLN metastasis as pN1 or pN2.

## Conclusions

As previously reported, bladder cancer metastasis limited to the PVLNs can occur. In the present study involving 115 bladder cancer patients undergoing radical cystectomy and pelvic lymph node dissection, we mainly demonstrated that: 1) pT stage at cystectomy was significantly higher in PVLN-positive cases than in pN0 cases; 2) the number of positive PVLN and the rate of extracapsular extension in the PVLN were not significantly different between the isolated PVLN-positive and concurrent non-PVLN-positive groups; and 3) PVLN metastasis was associated with a higher risk of mortality especially in those with concurrent other node-positive disease. Importantly, worse prognosis was observed in bladder cancer patients with isolated PVLN metastasis (vs. pN0 disease) and those with concurrent PVLN/non-PVLN metastases (vs. PVLN-negative/non-PVLN-positive disease). These outcome data may support the AJCC 8th edition bladder cancer staging system, while it possibly needs to be slightly amended for the classification of the PVLN as a regional node. Our findings also indicate the importance of diligent histopathological examination of radical cystectomy specimens to identify the PVLNs.

## Data Availability

Due to ethical restrictions, raw data underlying this study are available upon request to the corresponding author.

## Abbreviations

AJCC: American Joint Committee on Cancer
CI: confidence interval
HR: hazard ratio
non-PVLN: other pelvic lymph node
PVLN: perivesical lymph node
